# Are we testing visual acuity adequately?

**Published:** 2018-06-03

**Authors:** Eytan Blumenthal, Michael Mimouni

**Affiliations:** 1 Director, Department of Ophthalmology, Rambam Health Care Campus, Haifa, Israel.; 2 Resident, Department of Ophthalmology, Rambam Health Care Campus, Haifa, Israel.

**Visual field testing has benefited from advances in computerised technology and is considered highly accurate and reliable, whereas visual acuity testing still relies on the examiner to apply their best judgement. Is it time to reconsider?**

**Figure:**
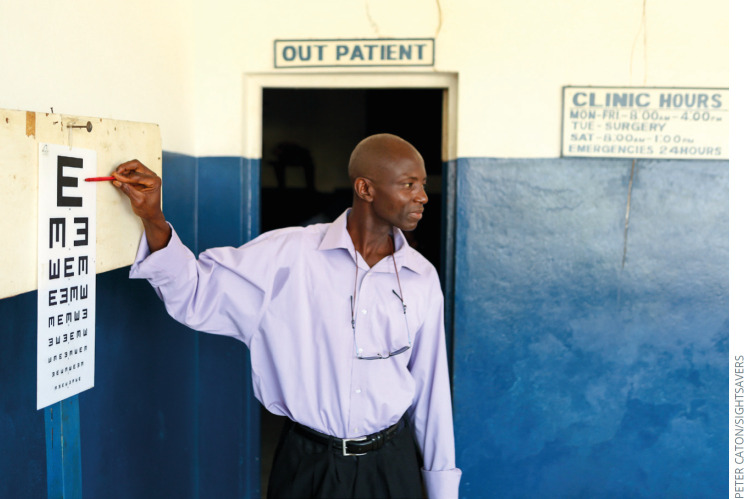
Visual acuity being tested using a tumbling E chart. SIERRA LEONE

Visual acuity (VA) is perhaps the single most important piece of information obtained during an eye examination. Great importance is attached to it, as well as to any change noted. VA was chosen to be a primary outcome measure in numerous clinical trials on macular degeneration, cataract surgery refractive surgery and others.

Therefore, VA testing should ideally benefit from the latest development in computerised technology and diagnostic algorithms. Is that really the case? While some may argue that the Early Treatment Diabetic Retinopathy Study (ETDRS) chart has revolutionised and standardised VA testing, one could wonder whether the ETDRS VA test methodology has more in common with a computerised visual field test or Goldmann perimetry.

**“Much can be learned from visual field testing.”**

In fact, much can be learned from visual field testing. Whereas VA and VF tests examine different aspects of the visual function, the progress made in the field of VF testing over the last 20 years might lead us to revisit the methodology used to test VA.

A typical automated 24–2 visual field test assesses the vision threshold in 52 locations, attaching a numerical value to each location. In contrast, a VA test checks the vision threshold in only a single location. Moreover, at each of the 52 locations used in a visual field text, the outcome is on a continuous scale, divided into 40 discrete steps (from 1 dB to 40 dB). Typically, VA is expressed as one of just 10 discrete steps (0.1 – 1.0), and the the standard ETDRS chart has 11 steps (11 lines span the 0.1–1.0 range). In VF testing, in order to achieve a more accurate estimation of the endpoint, the threshold is typically crossed multiple times, in opposing directions (from seeing to non-seeing, again to seeing, and back to non-seeing), whereas in VA testing the threshold is crossed only once.

In VF testing, each patient reply determines the brightness of the next stimulus projected, forming a dynamic, interactive test, whereas in VA testing the same questions are asked in the same order. In VF testing, the computer determines the final threshold value for each location, based on a complex algorithm, whereas in VA testing it is the examiner who relies on their best judgment. A VF test is not considered complete without reliability scores, whereas reliability and repeatability are not part of a VA test.

In visual field testing, during the course of a full-threshold 24–2 VF test, 8 VF locations are routinely checked twice, to measure repeatability. If so, why not routinely repeat VA measurements, say, 3–4 times? If a 24–2 SITA visual field test takes under 7 minutes, determining the final threshold value reliably some 52 locations, shouldn't a comparable VA test take under 1 minute? Hence, should a well-designed and executed computerised VA test take any longer than a manual Snellen or ETDRS VA test takes?

In addition, not all patients need to have their test start point at the 20/200 letter size. An improved algorithm may take into account previous documented VA measurements, the population the subject belongs to (i.e. a school screening test versus a retina clinic) and/or pre-test probability modelling.

After noticing these methodological differences between visual field and VA tests, we can ask why we so casually accept a testing procedure that does not stand up to other diagnostic procedures' standards. While it may be argued that variability in the subjective human response limit any potential benefits of incorporating more refined approaches, I believe that the progress made during the 20 years of automated VF testing serves as evidence contradicting this opinion. In fact, precisely because of the variability inherent in these subjective psychophysical tests, averaging multiple responses, as well as utilising thresholding algorithms, may allow more refined endpoints.

Others may argue that more refined VA results are of little clinical benefit, since VA is primarily used as a means to refract patients, and the smallest meaningful change in a prescription (1/4 spherical diopter) is roughly the equivalent of one Snellen chart line.

Following are several scenarios where our limited ability to test for VA accurately may sometimes get in the way of our goals. For example:

1. The enormous deterioration in vision that occurs when a patient's VA reduces from 20/200 to 20/400 is barely detectible using current methods.
2. More accurate VA data (with scores along a continuous scale (such as 20/32 vs. 20/37), along with reliability and confidence intervals, could increase the power of clinical trials, enabling a decrease in sample size, or alternatively, could shorten the duration of the study. With current methods, this is not possible.
3. We are unable to conveniently quantify low VA (such as 20/800 vs. 20/900) and the less-thanideal measurements of ‘finger-counting’ and ‘hand-motion’.

In summary, it might turn out to be worthwhile to question the methods we currently use to test visual acuity in both clinical and the research settings.

**Your contribution is welcome**

Using more expensive equipment to test VA in the same way as VF testing would be helpful in a scientific setting (as LogMar was intended to do), but something low cost and fit for purpose is required for everyday use. We would like to see that happen, and you can help! Using the ideas and principles discussed in this article, think about how VA testing can be made more accurate and more reliable using charts and equipment that are easily available in countries with limited resources.

Write to **admin@cehjournal.org** with a 400-word description of your idea/approach - the best suggestions will be published in a future issue of the *Community Eye Health Journal.* Photographs are very welcome. Explain to patients that their photograph will be published online and obtain their written permission (we need to see a copy of this). The editor's decision is final.

